# Supplementary material for: Iterative improvement in the automatic modular design of robot swarms
Source: PeerJ Comput Sci. 2020 Dec 7;6:e322. doi: 10.7717/peerj-cs.322 (PMC7924708; doi:10.7717/peerj-cs.322)
Supplement: Supplemental Information 3 [file peerj-cs-06-322-s003.zip › argos3/doc/api/standalone/a00506_source.html]

ARGoS: plugins/robots/generic/control\_interface/ci\_positioning\_sensor.cpp Source File


- Main Page
- Related Pages
- Namespaces
- Classes
- Files

- File List
- File Members

# plugins/robots/generic/control\_interface/ci\_positioning\_sensor.cpp

Go to the documentation of this file.

```
00001 
00007 #include "ci_positioning_sensor.h"
00008 
00009 #ifdef ARGOS_WITH_LUA
00010 #include <argos3/core/wrappers/lua/lua_utility.h>
00011 #endif
00012 
00013 namespace argos {
00014 
00015    /****************************************/
00016    /****************************************/
00017 
00018    const CCI_PositioningSensor::SReading& CCI_PositioningSensor::GetReading() const {
00019      return m_sReading;
00020    }
00021 
00022    /****************************************/
00023    /****************************************/
00024 
00025 #ifdef ARGOS_WITH_LUA
00026    void CCI_PositioningSensor::CreateLuaState(lua_State* pt_lua_state) {
00027       CLuaUtility::StartTable(pt_lua_state, "positioning");
00028       CLuaUtility::AddToTable(pt_lua_state, "position",    m_sReading.Position);
00029       CLuaUtility::AddToTable(pt_lua_state, "orientation", m_sReading.Orientation);
00030       CLuaUtility::EndTable(pt_lua_state);
00031    }
00032 #endif
00033 
00034    /****************************************/
00035    /****************************************/
00036 
00037 #ifdef ARGOS_WITH_LUA
00038    void CCI_PositioningSensor::ReadingsToLuaState(lua_State* pt_lua_state) {
00039       lua_getfield(pt_lua_state, -1, "positioning");
00040       lua_getfield(pt_lua_state, -1, "position");
00041       lua_pushnumber(pt_lua_state, m_sReading.Position.GetX());
00042       lua_setfield(pt_lua_state, -2, "x");
00043       lua_pushnumber(pt_lua_state, m_sReading.Position.GetY());
00044       lua_setfield(pt_lua_state, -2, "y");
00045       lua_pushnumber(pt_lua_state, m_sReading.Position.GetZ());
00046       lua_setfield(pt_lua_state, -2, "z");
00047       m_sReading.Orientation.ToAngleAxis(m_cAngle, m_cAxis);
00048       lua_getfield  (pt_lua_state, -2, "orientation");
00049       lua_pushnumber(pt_lua_state, m_cAngle.GetValue());
00050       lua_setfield  (pt_lua_state, -2, "angle");
00051       lua_getfield  (pt_lua_state, -1, "axis");
00052       lua_pushnumber(pt_lua_state, m_cAxis.GetX());
00053       lua_setfield  (pt_lua_state, -2, "x");
00054       lua_pushnumber(pt_lua_state, m_cAxis.GetY());
00055       lua_setfield  (pt_lua_state, -2, "y");
00056       lua_pushnumber(pt_lua_state, m_cAxis.GetZ());
00057       lua_setfield  (pt_lua_state, -2, "z");
00058       lua_pop(pt_lua_state, 4);
00059    }
00060 #endif
00061 
00062    /****************************************/
00063    /****************************************/
00064 
00065 }
```

---

Generated on 10 Jul 2018 for ARGoS by 
 1.6.1
